# Dasatinib-Loaded Topical Nano-Emulgel for Rheumatoid Arthritis: Formulation Design and Optimization by QbD, In Vitro, Ex Vivo, and In Vivo Evaluation

**DOI:** 10.3390/pharmaceutics15030736

**Published:** 2023-02-22

**Authors:** Mahipal Reddy Donthi, Ranendra Narayan Saha, Gautam Singhvi, Sunil Kumar Dubey

**Affiliations:** Department of Pharmacy, Birla Institute of Technology and Science, Pilani (BITS-PILANI), Pilani Campus, Pilani 333031, Rajasthan, India

**Keywords:** dasatinib, local delivery, emulgel, skin permeation, anti-inflammatory activity, Freund’s complete adjuvant

## Abstract

The current study aimed to develop a topical emulgel of dasatinib (DTB) for rheumatoid arthritis (RA) treatment to reduce systemic side effects. The quality by design (QbD) approach was employed to optimize DTB-loaded nano-emulgel using a central composite design (CCD). Emulgel was prepared using the hot emulsification method, and then the particle size (PS) was reduced using the homogenization technique. The PS and % entrapment efficiency (% EE) were found to be 172.53 ± 3.33 nm (0.160 ± 0.014 PDI) and 95.11 ± 0.16%, respectively. The nano-emulsion (CF018 emulsion) in vitro drug release profile showed sustained release (SR) up to 24 h. MTT assay results from an in vitro cell line study revealed that formulation excipients had no effect, whereas emulgel showed a high degree of internalization. Furthermore, emulgel treatment significantly reduced LPS-induced TNF-α production in RAW 264.7 cells. The spherical shape was depicted in FESEM images of optimized nano-emulgel (CF018 emulgel) formulation. Ex vivo skin permeation was significantly increased when compared to the free drug-loaded gel (FDG). In vivo data revealed that the optimized CF018 emulgel is a non-irritant and is safe. In terms of paw swelling, the FCA-induced arthritis model demonstrated that the CF018 emulgel reduced paw swelling percentage compared to adjuvant-induced arthritis (AIA) control group. Following clinical testing in the near future, the designed preparation could be a viable alternative treatment for RA.

## 1. Introduction

Rheumatoid arthritis (RA) is a chronic inflammatory autoimmune disease, which causes inflammation in joints, cartilage destruction, and bone loss [1]. Though no cure for RA has been reported, in the last two decades, medications decelerating the disease progression and preventing joint deformities have emerged as a long-term management therapy for RA [2,3]. Currently approved medications for the treatment of RA include non-steroidal anti-inflammatory drugs (NSAIDs), corticosteroids, disease-modifying anti-rheumatic drugs (DMARDs), and biologics [4]. Recent investigations disclosed that first-generation tyrosine kinase inhibitors (TKIs), including baricitinib, upadacitinib, and tofacitinib, have shown better therapeutic activity against RA [5,6]. Other TKIs, such as fostamatinib, dasatinib (DTB), imatinib, and nilotinib, have shown effectiveness against arthritis [7,8,9,10,11]. Herein, the current study focuses on DTB for the treatment of rheumatoid arthritis via topical emulgel delivery. 

DTB is a second-generation, multi-targeting TKI, inhibiting different tyrosine kinase receptors (TKRs) [12,13]. DTB also inhibits the discoidin domain receptors 1 (DDR 1) and 2 (DDR 2), as well as tumor necrosis factor-alpha (TNF-α) and interleukin 6 (IL6), the receptors and inflammatory cytokines that play crucial roles in the progression of RA [8,14,15,16]. Oral DTB (SPRYCEL tablet) exhibits severe side effects, including pulmonary hypertension, myelosuppression, gastrointestinal bleeding, and pleural effusions [17]. To avoid the above-mentioned side effects, topical administration of DTB could be a viable approach, as topical administration can improve the therapeutic efficacy, local effect, and decrease oral-associated side effects. Though topical preparations are advantageous to achieve localized effects and increase penetration into underlying layers of skin, the conventional topical applications also present some disadvantages, such as not being able to deliver the hydrophobic drug and the required concentration of drug at the target site, i.e., the joint region, while being extremely sticky and causing discomfort in the patient [18,19,20,21,22]. To address these limitations, an emulsion-loaded gel can be used to incorporate and deliver a hydrophobic therapeutic moiety via a topical route. Emulgels are dosage forms produced by combining emulsions and gels, possess high penetration capacity, elegance after application, and can easily be wiped off from the skin [23]. Emulgels for dermatological use have several advantages, including having thixotropic behavior, acting as an emollient, having a longer shelf life, high drug loading capacity, exhibiting controlled release, and improving stability [24,25,26]. 

The quality by design (QbD) model emphasizes a systemic approach in the development of pharmaceutical products. This approach starts with predefined specifications anchored on the product, process knowledge, and process control to assure the quality of the finished product [27,28]. In the present study, formulation variables were investigated using a QbD-based approach. The end product, emulgel of DTB, was developed using carbopol ETD 2020 as a gel-forming agent, peceol and geleol as oily vehicles, labrosol and capryol 90 as surfactants, and transcutol-P as a permeation enhancer. The developed formulations were evaluated for their cytotoxicity and inflammatory cytokine (TNF-α) expression on in vitro cell lines. Further, the formulation was studied for its ex vivo skin permeation, in vivo skin irritation, and pharmacodynamic activity by Freund’s complete adjuvant (FCA)-induced arthritis on Sprague Dawley (SD) rats. 

## 2. Materials and Methods

### 2.1. Materials and Reagents

Peceol, geleol, labrasol, capryol 90, and transcutol-P were received as generous gift samples from Gattefosse (Mumbai, India). Dialysis membrane with a molecular weight cut off 12,000–14,000 and membrane filters (0.22 μm) were purchased from Merck (India). All other laboratory chemicals were purchased from HiMedia (Mumbai, India).

### 2.2. Equipments

Wenser HD touch screen analytical weighing balance (Labman scientific instruments, Delhi, India) and electronic weighing balance (Shimadzu, Kyoto, Japan) were used to determine weights, and a waterproof pocket pH meter was used to determine pH values (Eutech Instruments Pvt Ltd., Singapore). A stability chamber for accelerated stability (MK scientific instruments, Delhi, India) and attenuated total reflection infrared spectroscopy (Bruker’s, Ettlingen, Germany) were used for drug-excipient compatibility study and as tests for DTB API identification. A bath sonicator (Labman scientific instruments, Chennai, India) was used for solubilizing the API and buffer salts. Ultra turrax variable speed homogenizer (IKA Pvt. Ltd., Bengaluru, India) was used for formulation preparation. Rheometer MCR 92 (Anton Paar India Pvt. Ltd., Gurgaon, India) was used for rheological behavioral assessment of the formulation. For drug permeation studies, a diffusion cell apparatus (Orchid Scientific, Nasik, India) was used. High-performance liquid chromatography (Shimadzu, Kyoto, Japan) for quantitative sample analysis, as well as a Zeta sizer (Malvern ZS, Malvern, United Kingdom), were used for the determination of size, poly dispersity index (PDI), and zeta potential.

### 2.3. Animals

Sprague Dawley (SD) rats of either sex were selected, weighing 150–250 g, and were acclimatized one week before the experiment. The standard laboratory conditions of 25± 2 °C with a light-dark cycle of 12 h were maintained, and a standard pellet diet with free access to water was allowed. All the necessary procedures were followed as per the institutional animal ethics committee (IAEC/RES/31/04), Birla Institute of Technology and Science (BITS-Pilani), Pilani campus, Rajasthan, India. The animal study was performed in accordance with the Committee for the Purpose of Control and Supervision of Experiments on Animals (CPCSEA) guidelines, and all the instructions were followed with a human approach.

### 2.4. Pre-Formulation Studies

#### 2.4.1. Solubility of DTB in Different Vehicles

The solubility of DTB in various oils and surfactants was determined using the saturation–solubility method. Excess DTB was added to 1 g of vehicle in a screw-capped vial, and the mixture was vortexed for 5 min using a cyclomixer. The mixture was placed for 48 h at 180 rpm and 25 ± 1 °C on a rotating shaker [29]. Further, the mixtures were centrifuged at 5000 rpm for 20 min, and the supernatants were filtered through a 0.22-micron syringe filter and diluted with methanol. The solubilized amount of DTB in each vehicle was analyzed using HPLC (Shimadzu LC-20AD, Japan) [30].

#### 2.4.2. Construction of Pseudoternary Phase Diagrams

Oil [peceol and geleol (70:30)], surfactant (labrasol), and co-surfactant (capryol 90) were screened based on data obtained from the solubility. Furthermore, the water titration method was used to generate the pseudoternary phase diagram in order to determine the micro-emulsion area [31]. Surfactant and cosurfactant mixture (Smix) were prepared in 1:1 and 2:1 ratios. After that, the 1:9 to 9:1 ratio of oil and Smix mixtures were prepared for 1 g. All of the prepared ratios were thoroughly mixed and titrated with water while continuously mixing, and any turbidity was inspected. The pseudoternary phase diagrams were plotted using Triplot 4.1.2 software based on the obtained percentage components.

#### 2.4.3. Drug-Excipient Compatibility Studies by DSC

DSC thermograms of pure DTB, individual excipients, and physical mixture were obtained using the Shimadzu DSC 60 model. About 4 mg of the sample was taken in an aluminum crucible using dry nitrogen as the inert gas. The heating range was programmed to 20–300 °C, with a heating rate of 10 °C/min for analyzing each sample [32]. 

### 2.5. Study Design

**Figure 1:**
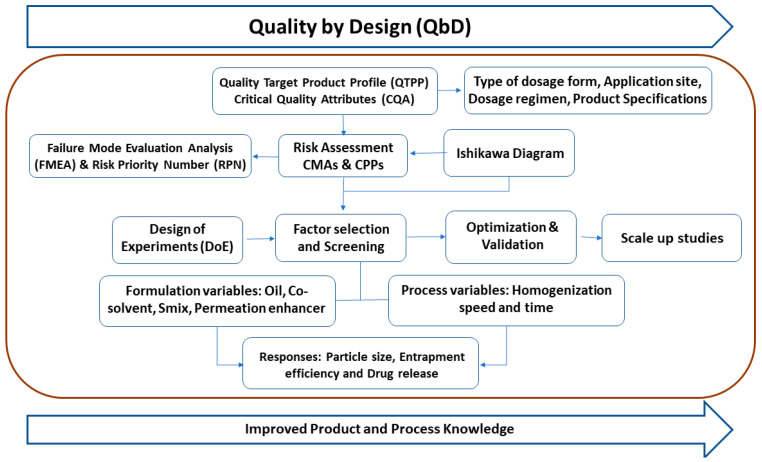
Representative image for steps involved in the QbD approach.

The experimental design offers a systematic, structured framework of the product process, as well as process knowledge and reliable information about the factors and their relative responses. Therefore, systematized execution is more crucial than ever. A representative study design is depicted as a route map in Figure 1.

#### 2.5.1. Quality Target Product Profile (QTPP) and Critical Quality Attributes (CQAs)

QbD promotes designing formulations for quality, rather than testing them. Quality control evaluation on manufactured products cannot achieve high quality without knowing the material type, the processes involved, or the quality attributes. The quality target product profile (QTPP) is a detailed description of the product’s safety and efficacy standards. Critical quality attributes (CQAs) were chosen to regulate the required features of the desired finished product in order to meet QTPP. The patient should be considered as the first priority while deciding the CQAs of the formulation. QTPP and CQAs were chosen based on research and knowledge of topical lipidic formulations before optimizing API-loaded nano-emulgel [33].

#### 2.5.2. Quality Risk Assessment

In the development of an emulgel formulation, critical formulation and process variables can alter the formulation’s critical quality attributes and cause formulation failure. In order to develop action plans for critical material attributes (CMAs) and critical process parameters (CPPs), the probability and potential severity of these risk factors, as well as the resulting failure modes, should be determined. An Ishikawa diagram was used to estimate the risk factors. For emulgel formulation, the Ishikawa diagram was used to identify the most influential factors in the formulation and process. In light of the potential factors that could affect the properties of the formulation, a risk-based matrix evaluation (REM) was carried out. The CMAs and CPPs were established by utilizing the REM to analyze the variables with low, medium, and high-risk potential. In order to conduct failure mode evaluation and analysis (FMEA), each factor’s severity, probability, and occurrence were assessed as showed in Table 1. The risk priority number (RPN) was determined using Equation (1), with a 0–10 ranking. The RPN score was used to identify CMAs and CPPs [34,35].

Severity (S) * Detectability (D) * Occurrence (O) = RPN (O)(1)

#### 2.5.3. Factor Screening Studies

The effect of oil, co-solvent (isopropyl myristate), Smix, and permeation enhancer (transcutol-P) on the size, entrapment, and drug release of the proposed emulsion were studied using a fractional factorial design (FFD) (2^4−1^), and the factors were selected based on the risk assessment report. The concentration of components keyed into two levels (low and high), i.e., oil concentrations of 5 and 20% *w*/*w*, Smix concentrations of 3 and 10% *w*/*w*, transcutol-P concentrations of 2.5 and 10% *w*/*w*, and isopropyl myristate concentrations of 2.5 and 10% *w*/*w* were used. Design-Expert software (version 7.0.0) was used to produce eight runs, at random, as shown in Supplementary Table S1, which served as the foundation for this study. Based on obtained data, the interaction and severity of the factors against the responses were depicted using Pareto charts. Further, the screened factors were carried out for the optimization studies. 

#### 2.5.4. Factor Optimizing Studies

Based on FFD data, oil and Smix were selected as the formulation variables, and the remaining two components (transcutol-P and isopropyl myristate) were kept constant in the formulation. In addition, the critical process parameters were selected from the Ishikawa diagram, i.e., homogenization speed and time. The selected formulation and process variables on the responses of size and entrapment were evaluated using a face-centered center composition design (CCD), without axil points, as shown in Table 2. The factors were keyed into two levels, i.e., low (−1) and high (+1). The design matrix suggested 17 experimental runs containing center points for each selected variable. 

#### 2.5.5. Optimization Data Analysis and Model Validation

Design-Expert software was used for the optimization, data analysis, and model validation to fit the experimental data into quadratic polynomial models for estimating the examined responses, as well as the inspected variables. The coefficient of correlation (R) and lack of fit analysis were chosen to serve as model assessment parameters from a variety of metrics. After that, the coefficients with *p* < 0.001 were considered when testing the analysis of variance (ANOVA) model. Furthermore, both three-dimensional response and two-dimensional counter plots were utilized in order to explore the factor response relationship. Based on the numerical optimization and desirability function, the best suggested composition was chosen. This is accomplished by switching the different CQA, i.e., desirable size, and maximizing the percentage entrapment of the drug in the formulation.

### 2.6. Formulation of DTB Emulsion

The formulation compositions are shown in Table 2. Each formulation was prepared as a single batch of 20 g. The formulation was fabricated using the O/W hot homogenization method, and the method of preparation is explained briefly in three steps, 1–3 [36,37].

Step 1: Oil phase preparation

The oil phase was prepared by dissolving the Smix in the oil and isopropyl myristate mixture. Further, DTB, MP, and PP were added to the above oil base, mixed well, and kept in a bath sonicator at 60 °C until the components were dissolved.

Step 2: Aqueous phase preparation 

The aqueous phase was prepared by dissolving the transcutol-P and sodium meta bisulphite and kept under stirring at 40 °C. 

Step 3: O/W nano emulsion preparation 

The oil phase was added dropwise to the aqueous phase under continuous homogenization at 40 °C. The mix was finally cooled down to room temperature.

### 2.7. Scale-Up Studies 

After optimizing the formulation based on QbD, the best formulation demonstrating desired responses was scaled up to the batch size of 50 mL and 100 mL. The quantities needed for formulation development were correspondingly increased with scale-up. The diameter of the batch vessel and the homogenizer were changed in the 50 and 100 mL size batches. The homogenizer with a 6 mm probe diameter (capacity 5–30 mL) was used to fabricate a batch of 20 mL, and the beaker surface was four times larger. The batch sizes of 50 and 100 mL were prepared using a homogenizer with a 20 mm probe diameter (capacity 30–250 mL). 

### 2.8. Characterization of Emulsion

#### 2.8.1. Attenuated Total Reflection-Infrared Spectroscopy (ATR-IR)

The IR spectrum of DTB, CF018 DTB loaded nano-emulsion (CF018 emulsion), and CF018 placebo nano-emulsion (CF018P emulsion) were determined using Bruker’s ATR, 4000 to 600 cm^−1^ with 0.5 cm^−1^ resolution.

#### 2.8.2. Measurement of Size, Polydispersity Index (PDI), and Zeta Potential (ZP)

Size, PDI, and ZP were measured using the dynamic light scattering (DLS) technique by a Malvern Zeta Sizer (Nano ZS, Malvern Instruments, UK) with a 173-degree angle of detection at 25 °C. About 100 µL of the emulsion was taken and diluted to 1ml (1:10 dilution) and analyzed for size, PDI, and ZP [38].

#### 2.8.3. Entrapment Efficiency (EE)

The EE was determined by separating the free drug present in the nanoemulsion using the dialysis method. Briefly, 1 g DTB-loaded emulsion was taken in a dialysis tube (3.5 kDa MWCO) and was closed by tying both ends. The dialysis bag was placed in 250 mL of distilled water at 37 ± 0.5 °C under stirring at 200 rpm. After an hour, 5 mL of the sample was aliquoted and analyzed using RP-HPLC. The EE of DTB in the emulsion was measured by using the following Equation (2) [39].

(2) $$\mathrm{EE}\;=\frac{\mathrm{Amount}\;\mathrm{of}\;\mathrm{DTB}\;\mathrm{dialyzed}}{\mathrm{Total}\;\mathrm{amount}\;\mathrm{of}\;\mathrm{DTB}\;\mathrm{in}\;\mathrm{formulation}}\times 100$$

#### 2.8.4. Thermodynamic Stability Studies

The optimized CF018 emulsion was subjected to allow various thermodynamic studies, such as a centrifugation test, freeze–thaw study, and heat–cooling cycle.

In the centrifugation test, the optimized CF018 emulsion (1 g) was diluted to 10 mL with water and allowed to undergo centrifugation at 5000 rpm for 15 min (REMI CPR-24) and assessed for instability. For the freeze–thaw stability, the temperatures were maintained to be −21 °C for 24 h followed by +25 °C for three cycles. Additionally, the formulation was subjected to three heating–cooling cycles. The temperature for the cooling cycle was 4 °C, and it was 45 °C for more than 48 h, as designed for the heating cycle [40].

#### 2.8.5. In Vitro Drug Release Study

The dialysis bag method was used to conduct in vitro drug release for selected emulsions and free drug solutions (FDS). In cellulose membrane, emulsion and FDS equivalent of 500 µg were taken (M.W cut off 12,000 KDa). The release medium was phosphate buffer pH 5.5, containing 1% triton X100, and the temperature was maintained at 37 ± 0.5 °C for 24 h, with continuous stirring at 400 rpm. A 1 mL sample was withdrawn at each predetermined time point and replaced with an equal amount of fresh medium to maintain sink conditions. The sample was analyzed by HPLC at 320 nm after suitable dilution. The drug release kinetics and mechanisms of optimized formulation were analyzed using the DD solver dissolution kinetic modeling software. The best fit was selected based on the obtained R^2^ values from each of the models [21].

#### 2.8.6. Morphology by Field-Emission Scanning Electron Microscopy (FESEM)

Field-emission scanning electron microscopy (AURIGA^®^ Carl Zeiss, Oberkochen, Germany) was used to investigate the morphology of nanoemulsion. A drop of CF018 emulsion was appropriately diluted with distilled water and placed on the surface of the coverslip and dried well. Before FESEM analysis, samples containing coverslips were coated with a thin layer of gold (10 nm). Images were captured at a 2 kV accelerating voltage [41].

### 2.9. Preparation of Emulgel

The emulgel was prepared using optimized emulsion formula with slight modifications in the aqueous portion for gelling agent addition, and the preparation process was explained briefly. The oil phase was prepared by dissolving the Smix in a peceol and isopropyl myristate mixture. Further, DTB, methyl paraben (MP), and propyl paraben (PP) were added to the oil base, mixed well, and kept in a bath sonicator at 60 °C until the components were dissolved. In the aqueous phase, 40% Milli-Q water was taken from the total water content designed in the formula and added to transcutol-P and sodium meta bisulphite, heated at 40 °C, and added dropwise to the oil phase to form the nano-emulsion under continuous stirring. The formed nano-emulsion was homogenized at 15,000 rpm for 7 min and cooled down to room temperature. An amount of 0.5% Carbopol ETD 2020 was added to the rest of the 60% Milli-Q water, and it was neutralized to pH 5.5, with 1% NaOH solution, to form a gel at room temperature under continuous homogenization. The preformed emulsion was added dropwise to the gel base under continuous stirring at room temperature to form the nano-emulsion-loaded gel known as nano-emulgel (CF018 emulgel) [36].

### 2.10. Evaluation of Emulgel

#### 2.10.1. Physical Appearance

The prepared DTB-loaded nano-emulgel was visually inspected for color, consistency, and homogeneity [42].

#### 2.10.2. pH Determination

The prepared CF018 emulgel pH was determined using calibrated hand pH meter. A concentration of 10% emulgel dispersion was prepared with Milli-Q water, and the measurement was recorded at ambient temperature [25].

#### 2.10.3. Spreadability Study

The spreadability of the optimized CF018 emulgel was performed using the graph paper method. Two known-weight glass slides were placed over the graph paper. x and y axes were displayed, and a square was drawn on chart paper. An amount of 0.5 g of gel was placed on the glass slide within the square. Then, the second glass slide was kept over the first slide, and the weights were added incrementally. The incremental diameter of the spread gel was noted [43].

#### 2.10.4. Drug Content Determination

The drug content in the formulation was evaluated by dissolving a known amount of CF018 emulgel in methanol using sonication. After suitable dilution, the sample was analyzed at 320 nm using HPLC. 

#### 2.10.5. Particle Integrity of Lipid Emulsion-Loaded Gel

The fabricated CF018 emulgel formulation was dispersed in Milli-Q water and thoroughly vortexed to obtain a uniform dispersion. Further, the PS of the dispersion obtained was evaluated using a Malvern Zeta Sizer. Additionally, the morphological characteristics of the dispersed particles were confirmed by FESEM.

#### 2.10.6. Rheology Study

##### Viscosity

The selected CF018 emulgel was evaluated for viscosity using an Anton Paar MCR 92 and a flat-faced spindle with a constant shear rate of 10 at 25 °C. the viscosity average of twenty points was noted. 

##### Shear Flow

This technique was used to determine the flow tendency of the selected formulation. Specifically, it aids in determining the dependence of viscosity on shear rate or shear stress. The continuous ramp step was selected with a shear rate range of 0 to 10 S^−1^ for 50 points at 25 °C. The results obtained were recorded as the shear rate, shear stress, and viscosity of each point [44].

##### Strain Sweep Test 

In this test, the formulation was characterized by increasing oscillatory strain (OS) at a constant frequency. The results are expressed in terms of the formulation’s storage (G′) and loss (G″) moduli as the strain range increases, which also provides information about the Newtonian behavior or linear viscoelastic region (LVR) of the formulation. Strain sweep measurements were taken at 32 °C in the range of 0.01 to 100%, with a constant angular frequency of 10 s^−1^.

##### Frequency Sweep Test (FST)

The FST is used to determine the relationship between testing frequency and a formulation’s storage (G′) and loss (G″) moduli. Furthermore, it provides information about a material’s viscoelastic properties and state by comparing the two G’ and G″ values over a frequency range.

### 2.11. In Vitro Cell Line Study 

#### 2.11.1. Cytotoxicity Studies

The cytotoxicity of the CF018 emulgel was examined using HaCaT (human keratinocyte) cell lines. The cell growth medium was composed of 90% Dulbecco’s modified eagle’s medium (DMEM) and 10% fetal bovine serum (FBS). The MTT [(3-(4, 5-dimethyl thiazolyl-2)-2, 5-diphenyltetrazolium bromide)] test was used to investigate cell cytotoxicity. The number of viable cells in the cell suspension was determined using a hemocytometer and the trypan blue exclusion method. For the test, 5 × 10^3^ cells/ well in a 96-well plate were seeded and placed into each well of a 96-well culture plate for 24 h in a CO_2_ incubator at 37 °C with 5% CO_2_ saturated humidity. The following day, medium was replaced with suspension of free DTB and CF018 emulgel prepared in 10 mL of DMEM medium. Each suspension was added at different concentrations of 50, 100, 150, 250, 500, 750, 1000, 1500, 2500, and 5000 nM. After 48 h, the medium containing the suspensions was discarded, followed by a PBS wash and an MTT test. To assess the viability of the cells, the absorbance was measured at 570 nm with a reference wavelength of 630 nm using an ELISA microplate reader (Biotek, Winooski, VT). The experiments were carried out in triplicate, and the results were expressed as the means ± SDs [45]. 

#### 2.11.2. Effect on the Production of TNF-α

This study examined how CF018 emulgel affected RAW 264.7 cells’ ability to produce TNF-α (isolated macrophages from mouse blood). DMEM (Dulbecco’s modified eagle’s medium; high glucose) + 2 mM glutamine + 10% FBS were used as cell supplement media. RAW264.7 cells were pre-incubated for 2 h with DTB, CF018 emulgel, and diclofenac sodium (DCS) after being seeded at a density of 2 × 10^5^ cells per well in 24 well plate. Lipopolysaccharide (1 µg/mL) was used to stimulate the cells for 24 h. A sandwich ELISA kit was used to determine the amount of TNF-α produced in cell supernatants according to the manufacturer’s recommended experimental procedures. The absorbance at 450 nm was measured using an ELISA microplate reader (Biotek, Winooski, VT, USA). The studies were performed in triplicate, and the data were presented as a mean ± SD.

### 2.12. Ex Vivo Studies

#### 2.12.1. Ex Vivo Permeation Study

Optimized CF018 emulgel formulation and the free drug-loaded gel (FDG) comparative permeation studies were conducted. The study was carried out in fabricated Franz diffusion cells (1.7 cm^2^ area and volume 30 mL), using rat skin. The skin was isolated and placed between the donor and acceptor compartment of Franz diffusion cells, placing the dorsal side upward. Phosphate buffer, pH 5.5, containing 1% Triton X100 as a release medium at 37 ± 0.5 °C under continuous stirring at 400 rpm was used throughout 24 h. A 1 mL sample was withdrawn at each predetermined time point and replaced with an equal amount of fresh medium. The sample was analyzed by HPLC at 320 nm after suitable dilution [46].

#### 2.12.2. Skin Deposition Study

The developed CF018 emulgel formulation was applied to the dermal surface of the skin. After 24 h, the skin was cut into small pieces and treated with extraction solvent (methanol), followed by homogenization for 20 min to extract the deposited drug embedded in the skin. Further, the extraction solvent was centrifuged at 5000 rpm for 10 min. The supernatant was collected, and the deposited drug content was analyzed using RP-HPLC [43,47].

#### 2.12.3. Bio-Adhesion Study

Rat skin freshly isolated was sectioned into pieces (2.5 cm^2^) and washed in phosphate buffer saline. Separately, the rat skin was tied to two slides. One slide was fixed to the top of the wooden platform, while another slide was tethered to the right side of the balance. After that, the left and right-side pans were balanced by the addition of extra weights to the left-side pan. Approximately 1 g of CF018 nano emulsion-loaded placebo gel (CF018P emulgel), CF018 emulgel, and 0.5% Carbopol gel formulations were placed in between two glass slides containing hairless rat skin, and the excess weight was removed from the left side pan to sandwich the two slides containing skin and to remove the entrapped air between them by applying pressure, and then it was kept in the same position for 5 min, and then water was added slowly to the left pan until the slides were separated. The weight (gram force) of the added water was calculated and noted as bio-adhesive strength. It was calculated using Equation (3) [20].

Bio-adhesive strength = Weight required (g)/Area (cm^2^) (3)

### 2.13. In Vivo Studies

#### 2.13.1. Skin Irritation Study 

The experiment was carried out according to the IAEC guidelines. One day before the beginning of this experiment, the hair on the rat’s dorsal side of the skin was removed with a shaver. The rats were divided into five groups (n = 3). Group I, untreated group, Group II, FDG-free DTB loaded gel (200 mg from 0.05% gel); Group III, CF018 emulgel (200 mg from 0.05% gel); Group IV, CF018P emulgel (200 mg from 0.05% gel); Group V, received 5% SLS gel. Finally, application areas were visually examined for erythema. Erythema severity was measured using the following scale, with scores ranging from 0 to 4. 0: no erythema. 1: Mild (barely perceptible light pink). 2: Severe (dark pink). 3: Mild to severe (light red). 4: Severe (extreme redness). Additionally, the images were taken at 0, 6, 12, 24, 48, and 72 h intervals [48,49,50]. 

Skin patches were assessed for histopathology. At the end of the 24 h, all of the animals were sacrificed. The skin biopsy (1 cm^2^) samples were stored in a 10% formalin solution for 48 h before being processed for histopathology studies. The specimens were prepared according to the Banchroft developed protocol [51].

#### 2.13.2. Assessment of the Anti-Inflammatory Effect 

The adjuvant arthritis model in rats was used to assess the CF018 emulgel therapeutic effect for RA. Rats weighing 150–250 g were divided into six groups at random. The following details were provided for each group: Group I, AIA-adjuvant induced arthritis (positive control); Group II, received MFD-marketed diclofenac formulation (0.5 g/kg); Group III, received CF018 emulgel (200 mg containing 100 µg dose); Group IV received a CF018P emulgel; Group V received FDG (200 mg containing 100 µg dose); Group VI, normal group (negative control), and 0.1 mL of FCA was injected to the hind paws of rats, except group VI. The volume of hind paw was measured using a water plethysmometer at 0, 0.5, 1, 2, 3, 6, 9, 12, 15, 18, 21, 24, and 27 days after FCA injection, and, at the pinnacle of the swelling region of the joint, it was treated with respective formulations (from day 5). The effectiveness of the anti-inflammatory treatment was evaluated by swelling degree using Equation (4).

(4) $$S\left(\%\right)=\frac{\mathrm{Vt}-\mathrm{Vn}}{\mathrm{Vn}}\times 100$$

where, Vn—paw volume before the injection of FCA; Vt—paw volume at day t.

#### 2.13.3. Assessment of Arthritis from Physical Parameters

During the test period, between 0 and 27 days, the rats’ body weight (% increase in body weight) and arthritis scores (based on visual inspection) were recorded every three days [52].

#### 2.13.4. Nociceptive Threshold

The nociceptive threshold was determined using Eddy’s hot plate method [53]. The rats were acclimatized three days before the experiment. For all animals, the pain threshold was determined by the paw withdrawal latency (PSL) to radiant heat stimuli (paw withdrawal) with a maximum cut-off time of 15 s.

#### 2.13.5. Motor Incoordination Test

The motor coordination test was performed on arthritis rats treated with topical CF018 emulgel formulation using an accelerating rota rod [54]. Rats were positioned on the rotating rod of the apparatus, and the time it took for each rat to fall from the roller was noted. Prior to the data collection day, all the animals were acclimatized to the accelerating rota rod over three training sessions. The test was conducted on days 0, 0.5, 1, 2, 3, 6, 9, 12, 15, 18, 21, 24, and 27 of the arthritis study. Each animal in each group had three trials at 10 min time intervals, and the fall time was recorded.

### 2.14. Statistical Analysis

Each experiment was repeated three times, and the results are provided as the mean ± SD. Statistical analysis was executed using two-way analysis of variance (ANOVA), followed by Sidak’s multiple comparison tests estimated using GraphPad Prism (v 7.0), *p* values < 0.05.

## 3. Results and Discussion

### 3.1. Solubility Studies

Solubility of DTB was determined in various vehicles, as depicted in Supplementary Figure S1. Oils, such as labrafac lipophile, misine CC, labrafac PG, and peceol were evaluated. Surfactants, such as capryol 90, labrasol, labrafil M2125, labrafil 1944, and labrasol ALF were included and based on the maximum solubility of the drug. Peceol as the oily vehicle, labrasol as the surfactant, and capryol 90 as the cosurfactant were selected for further studies. Peceol was found to have maximum solubility of 3.06 ± 0.06 mg/g. Labrasol ALF had a solubility of 10. 61 ± 0.03. Further, capryol 90 had a solubility of 8.76 ± 0.07 mg/g.

### 3.2. Pseudoternary Phase Diagrams

**Figure 2:**
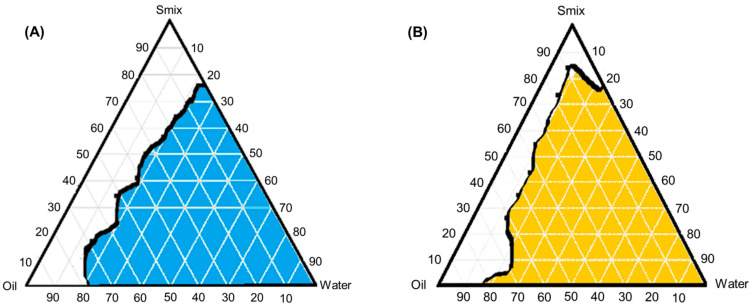
Pseudoternary phase diagrams series. (**A**) Containing oil and Smix ratios of 1:1. (**B**) Containing oil and Smix ratios of 2:1, blue and orange colors (emulsion zone), white color (non-emulsion zone).

The pseudoternory diagrams were plotted to spot the emulsion area, Figure 2. Here, oil [peceol and geleol (70:30)], surfactant (labrasol), and cosurfactant (capryol 90) were selected. Ratios of 1:1 and 2:1 surfactant mixtures were prepared, as well as oil and Smix ratios of 1:9 to 9:1. Mixtures were prepared and titrated with water until turbidity was seen. In both ratios, a 2:1 ratio of Smix had more emulsion area. Hence, a 2:1 ratio of Smix was used for designing the emulsion formulation.

### 3.3. Differential Scanning Calorimetry (DSC)

**Figure 3:**
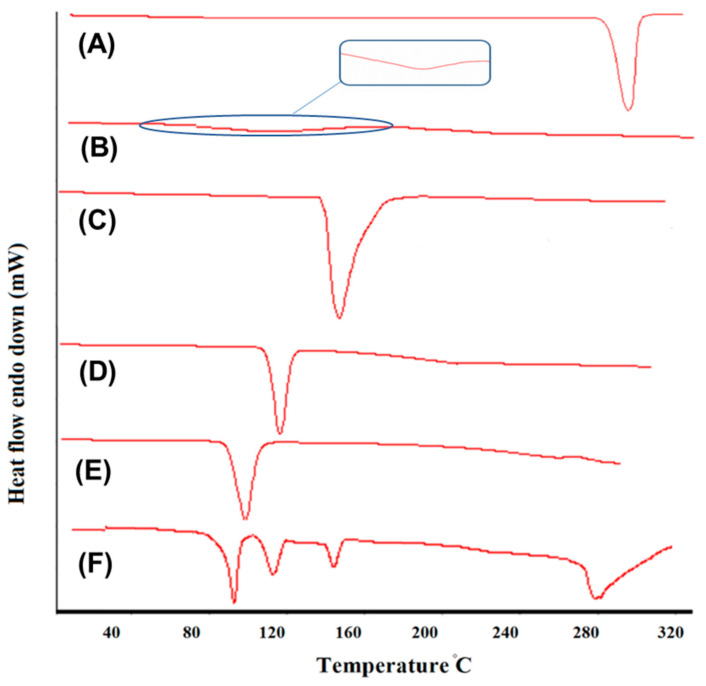
DSC thermogram of (**A**) DTB; (**B**) Carbopol ETD 2020; (**C**) sodium meta bisulphite; (**D**) methyl paraben; (**E**) propyl paraben; (**F**) physical mixture.

The pure drug, excipients, and physical mixture DSC were performed, as in Figure 3. DTB pure drug showed a sharp endothermic peak at 282.4 °C. Sodium meta bisulphite and MP and PP excipients showed sharp endothermic peaks at 154 °C, 124 °C, and 96 °C, respectively. Additionally, Carbopol ETD 2020 showed an endothermic glass transition peak at 113 °C. In physical mixture, the melting point of DTB was found to be 283.1 °C. Thus, there was no interaction between drug and excipients. Further, the excipients were used in the formulation.

### 3.4. Identification of QTPP and CQAs

After identifying the QTPP, the QbD method for emulgel preparation was developed. Supplementary Table S2 shows the QTPP that was selected for DTB-loaded emulgel. The CQAs were preferred over the QTPP because they were explored to affect the final product characteristics. Supplementary Table S3 shows that PS, EE, and drug release were identified as CQAs of DTB-loaded emulgel and have more impact on product quality.

### 3.5. Risk Identification and Risk Assessment

**Figure 4:**
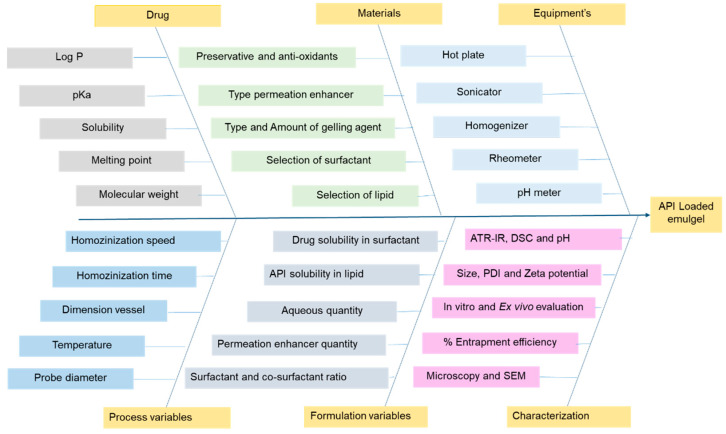
Ishikawa diagram illustrated the potential CMAs and CPPs that effect the CQAs of API-loaded emulgel formulation.

The risk assessment was conducted, and CPPs and CMAs were identified using the Ishikawa diagram shown in Figure 4. Based on reported studies and preliminary results, REM was formulated. The RPN scores were assigned in FMEA based on the risk potentiality; the scores were given based on reported studies. Table 1 shows the risk estimation matrix and RPN scores of the CMAs and CPPs. RPN scores greater than 125 are considered as vital for optimizing critical attributes. The lipid and Smix concentrations can influence the drug loading and particle size of emulsion, which can affect the drug permeation and efficacy of the formulation, and are hence considered as high risk parameters. Further, homogenization speed and time were considered as potential risk factors, as they may affect particle size and uniformity of particle distribution. 

### 3.6. Factor Screening Studies

CMAs from risk assessment were used in the screening studies to analyze the interaction between factors and responses. For formulation screening, a two-level factorial design (Design-Expert 7.0.0) with eight trials was used with four factors at low- and high-level concentrations. The suggested amounts of lipid, co-solvent, permeation enhancer, and Smix concentration were investigated. Eight batches were carried out in accordance with the design, as shown in Supplementary Table S1. Formulations were designed by varying independent variables while carrying all other parameters constant (process and formulation parameters), and PS, EE, and drug release were investigated as dependent variables. The concentrations of the preservatives MP and PP used were 0.5% and 0.05% (*w*/*w*), respectively, and the anti-oxidant sodium metabisulphate (0.2%) remained constant across all formulation trials. The interaction and efficiency of the factors against the responses were represented in the form of a Pareto chart, as seen in Supplementary Figure S2, and the percent contribution of the factors against the responses was depicted in Supplementary Table S4. Supplementary Table S5 shows the response values obtained after implementing a two-level factorial design with four factors and two levels.

### 3.7. Factor Optimizing Studies

The report resulting from the screening studies finalized various critical material attributes, such as lipid and Smix, as well as critical process parameters, such as homogenization time and speed, based on the risk assessment report. PS and EE are considered as CQAs in the responses.

For formulation optimization, a CCD with 17 trials was keyed with four factors and three levels using DoE. This suggested quantity of lipid, Smix concentration, homogenization speed, and time were investigated at three levels: low, medium, and high. In accordance with the design, 17 formulations were prepared, as shown in Table 2. Formulations were developed by varying independent variables while carrying all other parameters constant (process and formulation parameters), and PS and EE were studied as response variables. The concentrations of co-solvents, permeation enhancers, preservatives, and antioxidants used in all trials were constant. Analysis of variance was used to assess the model efficiency and the significance of the selected input variables. The central composite design (CCD) with four components and three levels was used, and the response values obtained after the design was executed are shown in Table 2.

### 3.8. Independent Variables Effect on PS

**Figure 5:**
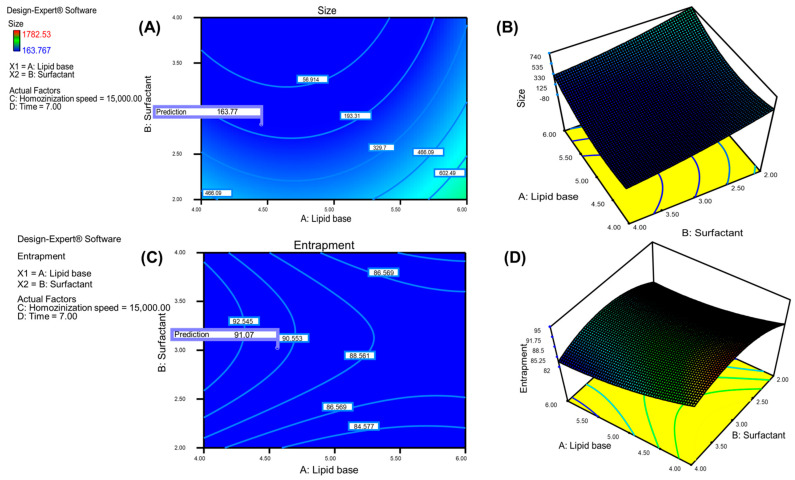
CCD optimization graphs illustrating the effect of independent variables PS and EE. (**A**) The contour plot graph of factors effecting on PS. (**B**) The three-dimensional graph of factors effecting on PS. (**C**) The contour plot graph of factors effecting on EE. (**D**) The three-dimensional graph of factors on effecting EE.

The PS of the API-loaded emulsion after 17 trials were in the range of 163.77–1782.53 nm, as shown in Table 2. The quadratic model has a 229.45 *F*-value, indicating its significance. Figure 5A,B demonstrate the contour plots and three-dimensional plots of the response variable, PS. When compared to the pure error, the lack of fit was found to have a F-value of 2, which indicates its insignificance. The coded values of chosen independent variables are depicted in regression, Equation (5).

Size = 271.12 + 145.00A − 1.20B − 40.33C − 68.43D + 21.48AB − 30.70AC − 44.77AD − 344.55BC − 191.84BD + 322.59CD + 205.15A^2^ + 48.62B^2^+ 60.22C^2^ − 10.38D^2^ (5)

The collusive effect is represented by the positive symbol in the regression equation, which denotes an increase in response value with respect to the corresponding input variable. The hostile effect is represented by the negative symbol in the regression equation, which indicates a decrease in response value with the corresponding input variable. The lack of fit suggests dissimilarity in the best fit model’s data and allows the model fitting of experimental results to be competent. The model’s best fit is indicated by an insignificant lack of fit in the experimental results. The regression, Equation (5), represents the increase in PS as lipid concentration increases. The PS was reduced with an increase in percent Smix concentration, homogenization time, and speed. The Smix has the potential to reduce surface tension at the oil–water interface and improve particle stabilization, resulting in smaller particles. The increased speed and time of homogenization reduces the PS of the formulation, breaking the large particles into smaller size particles.

### 3.9. Effect of Independent Variables on EE

The average EE of the API-loaded emulsion after 17 trials was in the 84.31–95.23% range, as shown in Table 2. The quadratic model’s F-value of 711.82 indicates the model’s significance. Figure 5C,D revealed the contour plot and three-dimensional graph of the response variable EE. The lack of fit was insignificant, with a 2.21 F-value compared to the pure error. The regression, Equation (6), depicts the coded values of chosen independent variables.

Entrapment = 89.89 − 1.60A + 0.96B − 0.77C − 1.99D − 0.86AB − 1.84AC − 0.42AD − 0.37BC − 1.31BD + 2.46CD + 1.80A^2^ − 4.56B^2^ + 0.46C^2^ − 0.037D^2^ (6)

The regression, Equation (6), represents the increase in entrapment with an increase in concentration of lipid and Smix. The reduced EE was observed as the homogenization time and speed increased. The lipid and Smix concentrations increase drug solubility, which leads to an increase in entrapped efficiency. Increased homogenization time and speed decreased entrapment, as expected due to drug expulsion during the formulation’s breakage of micro particulates into nano size particles. 

### 3.10. Validation of the Design to Select Optimized Batch

**Figure 6:**
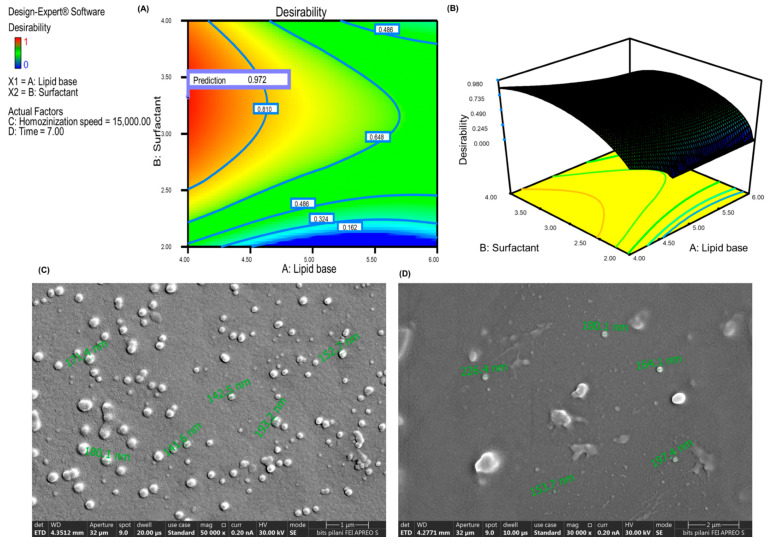
CCD desirability graphs illustrating factors against desirability value. (**A**) The contour plot graph. (**B**) The three-dimensional graph. (**C**) Morphological characterization FESEM image of CF018 emulsion. (**D**) The FESEM image of CF018 emulgel.

The numerical and desirability methods were used to optimize the API-loaded emulsion. The goal was to find the formulation with the best EE and smallest PS. Table 2 illustrates a summary of the requirement criteria that must be met in order to attain the anticipated response. The Design-Expert software produced 27 solutions, and the one with higher desirability close to 1 was picked for preparing the API loaded nano-emulsion (CF018 emulsion). The design was validated using the following formulation with lipid content (4%), Smix concentration (3%), homogenization speed (15,000 rpm), and time (7 min). The formulation’s mean PS was 172.53 ± 3.33 nm, with a PDI of 0.160 ± 0.014. The EE of the optimised formulation was found to be 95.11 ± 0.16 percent. The formulation’s percent drug release was greater than 89.90 ± 6.25 in 12 h, which represents a sustained release profile. The predicted values of EE and PS was close to the actual value, indicating the desirability of the design. Figure 6A,B depicts the desirability graphs and three-dimensional surface graphs of PS and EE for optimized formulation, and this same formulation was carried out for the scale-up studies.

### 3.11. Scale-Up Studies 

The optimized CF018 emulsion was selected for the scale up studies. The batch sizes of 20, 50, and 100 mL were formulated using the design space suggested parameters. The formulation compositions remained unchanged, but the amount of lipid and Smix concentrations were increased with respect to batch size. In the process parameters (PPs), the homogenization speed was constant for three batches, but the processing time was increased by 2 min (total 9 min) for 100 mL batch formulation. The 20- and 50-mL batch sizes were prepared, as per the design space suggested time (7 min). Additionally, the vessel diameter played a vital role in the size reduction process during homogenization. Thus, the homogenization time was adjusted according to the batch size, and also a suitable vessel size was selected with respect to the individual bath size.

### 3.12. Characterization of Emulsion

#### 3.12.1. Attenuated Total Reflection Infrared Spectroscopy (ATR-IR)

The IR spectra of DTB, CF018P emulsion, and CF018 emulsion are shown in Supplementary Figure S3. The characteristic peaks of DTB were found to be 1498.59 cm^−1^, 1581.11 cm^−1^, 1624 cm^−1^, 3229.9 cm^−1^, and 3402.12 cm^−1^ for C–H, C–C, C=O, O–H, and N–H (secondary amine N-H stretch), respectively. The distinct DTB peaks vanished in the CF018 emulsion, and the peaks that remained matched the CF018P emulsion. This indicates that the pure drug was entrapped within the lipid matrix, resulting in the loss of drug characteristic peaks.

#### 3.12.2. Measurement of PS, PDI, ZP, and EE

The PS of the three formulations were 172.53 ± 3.33 nm (0.160 ± 0.014 PDI), 167.90 ± 10.68 nm (0.225 ± 0.121 PDI), and 154.90 ± 11.09 nm (0.30 ± 0.07 PDI), respectively, for batches of 20, 50, and 100 mL. The average zeta potential of the optimized formulation was –57.233 ± 4.97 mV, and the zeta potential ranging from 40 to 60 mV is considered to be stable. Zeta potential was 40–60, which reduces particle coalescence/aggregation [55]. For 20-, 50-, and 100-mL batches, EE was 95.11 ± 0.16%, 95.25 ± 0.245%, and 95.42 ± 0.18%, respectively. According to the findings, all batches were found to have similar characteristics. The formulation morphology was observed to be spherical and in the 140–200 nm range, as shown in Figure 6C. The reported studies suggest that the PS of nanoformulation less than 200 nm exhibit improved permeation [56]. A PDI less than 0.300 indicates that the formulation is uniform.

#### 3.12.3. Thermodynamic Stability Study

The prepared emulsion was evaluated for thermodynamic stability and found to be stable when subjected to centrifugation and a freeze–thaw cycle. As a result of centrifugation and a freeze–thaw cycle, the nano-emulsion showed no signs of drug precipitation or creaming/cracking. Thus, this indicated its stability.

#### 3.12.4. In Vitro Drug Release Studies 

**Figure 7:**
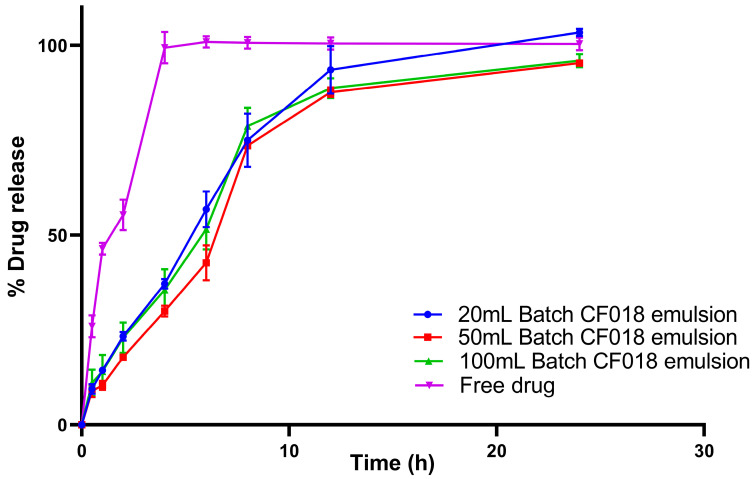
In vitro comparative drug release profile of CF018 emulsion (20, 50 and 100 mL) and free drug.

The in vitro release study of all three batches of 20, 50, and 100 mL CF018 emulsion and FDS were performed for up to 24 h, represented in Figure 7 Within 4 h, the FDS was found to show 100% drug release. The drug-loaded emulsion has shown drug release for up to 24 h. The drug release for 20-, 50-, and 100-mL batches after 6 h were around 56.78 ± 4.71%, 42.69 ± 4.62%, and 51.50 ± 5.32%, and, after 12 h, the release was 93.51 ± 6.25%, 87.63 ± 1.22%, and 88.71 ± 2.61%; after 24 h, the release was 103.40 ± 0.98%, 95.29 ± 0.38%, and 95.93 ± 1.73%, respectively. All CF018 emulsion batches illustrated SR patterns with no significant variation. The in vitro release profiles of the drug from all three batches (20, 50, and 100 mL) followed the Hixson Crowell model because the plots seemed to have the highest R^2^ values, i.e., 0.9912, 0.9710, and 0.9809, respectively. The obtained values were shown in Table 3. The model assumed that the rate of drug release is limited by dissolution and not diffusion, which indicates that the drug dissolved in the lipid might be responsible for the SR [57].

#### 3.12.5. Morphology by Field–Emission Scanning Electron Microscopy (FESEM)

The particles were found to be evenly distributed and of uniform size in the morphological study (Figure 6C). Additionally, they were non-aggregated, spherical, and had a smooth, flexible boundary, demonstrating their stability against Ostwald ripening due to specific collapse particles.

### 3.13. Emulgel Characterization 

#### 3.13.1. Physical Appearance, pH Determination, Spreadability, and Drug Content Determination

**Figure 8:**
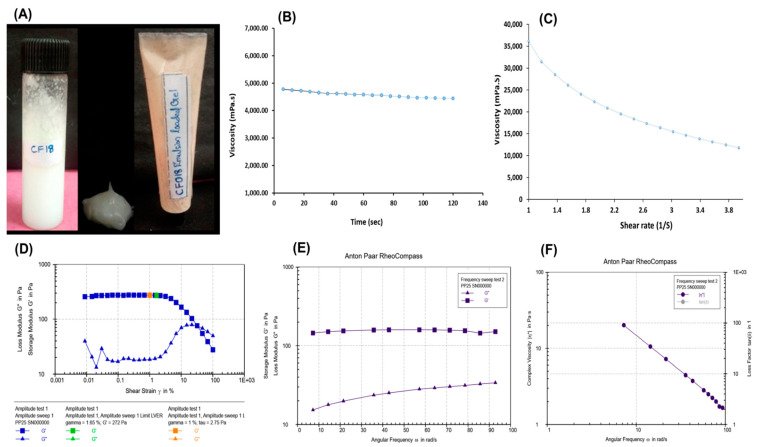
Rheological characteristics of CF018 emulgel. (**A**) CF018 emulsion and CF018 emulgel formulation. (**B**) Viscosity. (**C**) Shear flow. (**D**) Amplitude sweep test of formulation employing angular frequency. (**E**) FST of CF018 emulgel (loss modulus and storage modulus). (**F**) FST of CF018 emulgel (complex viscosity).

The prepared CF018 emulgel formulation had a milky white creamy appearance with a smooth homogeneous texture and excellent consistency (Figure 8A). Topical formulations should have a pH that is compatible with skin pH. A change in pH can irritate or disrupt the skin. Thus, pH of the formulation was adjusted to 5.5 ± 0.1 with 1% NaOH solution. The spreadability of the optimized emulgel formulation and placebo was found to be 1.20 ± 0.003 g/cm^2^ and 1.26 ± 0.05 g/cm^2^, respectively. No significant change was observed in drug-loaded formulation compared to the placebo gel. The drug content was found to be 98.25 ± 1.2%

#### 3.13.2. Particle Integrity in Hydrogel Matrix

The prepared CF018 emulgel was characterized for PS, and its PS was found to be similar to that of the emulsion, i.e., 170.01 ± 30.26 nm. Morphological analysis of the emulgel by using FESEM also showed similarity with that of the CF018 emulsion, as seen in Figure 6D. Henceforth, the data revealed that the nanocarriers remain intact after being loaded into the Carbopol ETD 2020 hydrogel matrix.

#### 3.13.3. Rheological Behavior

##### Viscosity

The average viscosity of the optimized formulation was determined to be 4580.13 ± 103.0232 mPa at a constant shear rate and at increasing time at constant shear rate, therefore showing no significant change in viscosity. Hence, Newtonian behavior of the gel is demonstrated, as shown in Figure 8B. 

##### Shear Flow

The flow characteristics of the CF018 emulgel was conducted, and the results were shown in Figure 8C. Here, the viscosity and shear stress varied with the function of shear rate. As a result, the formulation demonstrated non-Newtonian behavior that corresponded to the Herschel-Bulkley model, Equation (7).

σ = σ_0_ + K·γ^n^ (7)

where σ_0_ represents yield stress, K represents consistency index, and n represents power index; n < 1. 

The viscosity decreased as the shear rate increased, indicating shear thinning and pseudoplastic behavior of the CF018 emulgel. 

##### Strain Sweep Test

An oscillation strain sweep (OSS) test was used to find the linear viscoelastic region (LVR), in which the viscoelastic moduli change with time (or) frequency. The LVR ended with a 10% decrease (1.65%) in the storage modulus (G′) plateau value. Subsequently, the loss modulus (G″) started to go up. This meant that the viscoelastic properties might not extensively be independent of the strain amplitude, which indicates that the sample lost its original structure upon the function of oscillation strain (OS). The flexible long straight chain of polymers may form some coiling, which could lead to some degree of interlocking to reach a state with the least amount of energy. Furthermore, hydrogen bonding can also develop an infinite number of bridges between neighboring molecules, resulting in complete cross-linking. When OS was applied, polymeric chains uncoiled and aligned with the flow.

The polymeric chain state changed from disorderly to orderly, arranged during oscillation, and the transformation was reversible. In Figure 8D, the values of log G′ and log G″ versus the logarithm of % strain showed that the LVR had a predominant elastic behavior, indicating the strongest gel structure. 

##### Frequency Sweep Test 

When the gel’s structural integrity is intact, frequency changes do not affect G′ and G″ [58]. Figure 8E,F depicts the frequency distribution versus frequency logarithm. Elastic and viscous modulus were frequency independent in CF018 emulgel. G′ was greater than G″ across the frequency range, indicating strong gel structure.

To summarize the rheology, the viscosity study demonstrated an independent effect upon an incremental shear time at constant shear stress, indicating that the gel has an excellent consistency. The prepared emulgel showed a storage modulus greater than loss modulus G″ in frequency strain and frequency sweep tests, indicating a strong gel structure. The structural integrity of the gel formulation was indicated by the viscosity and frequency strain and frequency sweep tests. In addition, increasing shear stress with time transformed the solid gel state to a flowable state (shear thinning behavior), indicating spreadability. During topical application, the desired spreadability forms a thin layer on the skin’s surface with improved bio-adhesive properties, resulting in sustained release with an enhanced therapeutic effect. Hence, it can be concluded that DTB emulgel would be a viable option for the patient [59,60]. 

### 3.14. In Vitro Cell Line Study

#### 3.14.1. Cytotoxicity Studies

**Figure 9:**
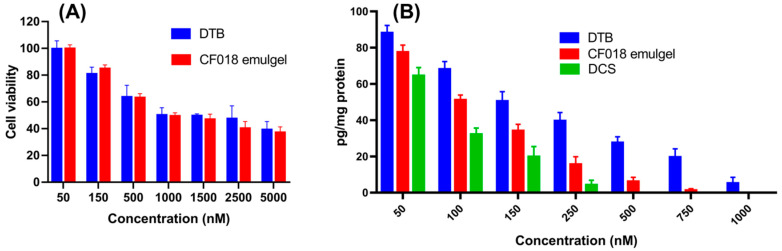
In vitro cell culture studies. (**A**) Cell viability study. (**B**) TNF-α expression level.

The cytotoxicity test for free DTB and CF018 emulgel was performed on HaCaT cell lines (immortalized human keratinocyte). Keratinocytes were chosen because they account for 95% of epidermal skin cells. The cytotoxic effects of free DTB and CF018 emulgel dispersion on HaCaT cell lines are shown in Figure 9A. DTB had no significant effect on keratinocyte cells. More than 50% cell viability was observed at a treatment of 1000 nM concentration of free DTB and CF018 emulgel. The findings demonstrated that excipients used in formulations are not toxic to keratinocyte cells.

#### 3.14.2. Effect on the Production of TNF-α

TNF-α plays a crucial role in the growth of fibroblasts and the induction of pro-inflammatory mediators including PGE2 and IL-1 during the pathogenesis of RA. As a result, it encourages synovitis and drives the destruction of bone and cartilage. As compared to free DTB, CF018 emulgel treatment significantly decreased the LPS-induced TNF-α production from RAW 264.7 cells, as depicted in Figure 9B. The results suggested that the anti-arthritic properties of the CF018 emulgel may inhibit inflammatory cytokines such as TNF-α. Inhibiting TNF-α production is associated with decreased inflammation and cartilage deterioration, which can halt the progression of arthritis.

### 3.15. Ex Vivo Studies

#### 3.15.1. Ex Vivo Skin Permeation Study

**Figure 10:**
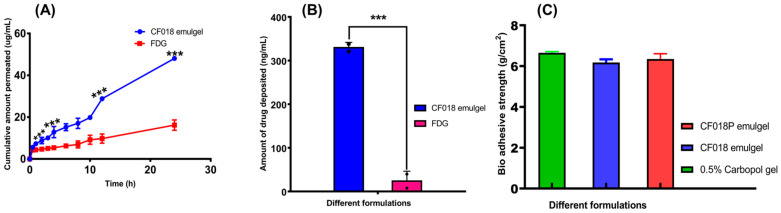
Ex vivo characterization of CF018 emulgel and DTB loaded gel. (**A**) Ex vivo permeation study of CF018 emulgel and FDG. (**B**) Skin deposition study. (**C**) Comparative bio-adhesion strength. *** *p* < 0.001 vs. FDG.

The optimized CF018 emulgel and FDG formulations were used for ex vivo permeation studies on rat abdominal skin using a pH 5.5 phosphate buffer containing 1% Triton X100 as release media, and the total amount of permeation over a 24 h period is shown in Figure 10A. After 12 h, the amount of drug permeated through the skin was found to be 28.42 ± 0.65 µg/mL and 9.78 ± 3.99 µg/mL for CF018 emulgel and FDG formulations, respectively. After 24 h, it was found to be 53.68 ± 0.24 µg/mL and 15.57 ± 4.49 µg/mL. The flux across skin was found to be 2.11 ± 0.25 µg /cm^2^ /h^−1^ for CF018 emulgel and 0.6 ± 0.19 µg /cm^2^ /h^−1^ for FDG formulations. CF018 emulgel has greater drug permeation (*p* < 0.001) and flux across the skin than FDG. Hence, the study concluded that the CF018 emulgel has higher permeation efficiency compared to FDG.

#### 3.15.2. Skin Deposition Study

After 24 h, the amount of drug deposited was found to be 326.03 ± 53.72 ng and 39.75 ± 7.65 ng for CF018 emulgel and FDG formulation, respectively, as shown in Figure 10B. The deposition was found to be 10-fold higher (*p* < 0.05) in the case of the CF018 emulgel as compared to the FDG. As a result, the study concluded that the CF018 emulgel has better deposition capacity and will deliver the drug to deeper layers in a sustained manner.

#### 3.15.3. Bio Adhesion Studies

The adhesive strength of CF018 emulgel, CF018P emulgel, and 0.5% carbopol plain gel was determined to be 6.1 ± 0.14, 6.35 ± 0.25, and 6.65 ± 0.05, respectively, Figure 10C. The adhesiveness of the CF018 emulgel was shown to have similar results to the placebo gel, but a slight variation (non-significant) was observed with 0.5% carbopol gel, indicating that the lipid nanoparticles dispersed uniformly in the aqueous gel base did not interfere with the swelling mechanism of the carbopol gel, Figure 10C [43].

### 3.16. In Vivo Studies

#### 3.16.1. Skin Irritation Studies

**Figure 11:**
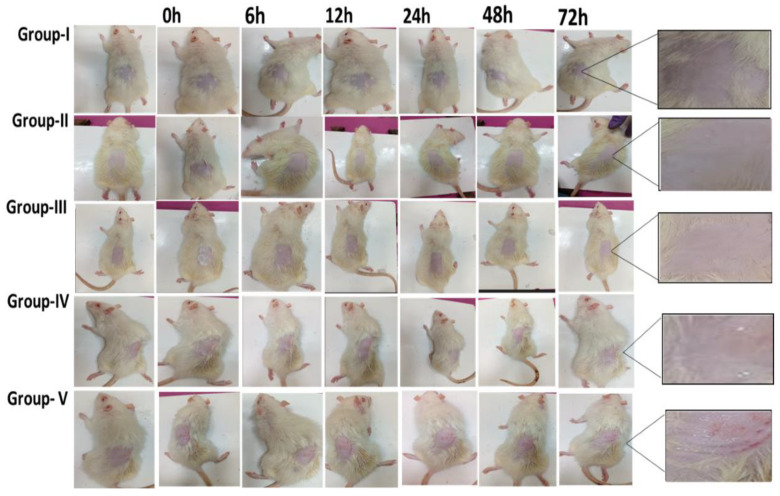
In vivo skin irritation study of Group I—untreated group; Group II—FDG (200 mg from 0.05% gel); Group III—CF018 emulgel (200 mg from 0.05% gel); Group IV—CF018P emulgel (200 mg from 0.05% gel); Group V—received 5% SLS gel.

After 72 h of observations, the scores for erythema, atonia, and fissuring of the five groups were represented in Supplementary Table S6. Except 5% SLS gel, all other formulations behaved similarly to the non-treated (negative control group). Therefore, the developed topical formulation is safe for the skin, and the study images were represented in Figure 11.

**Figure 12:**
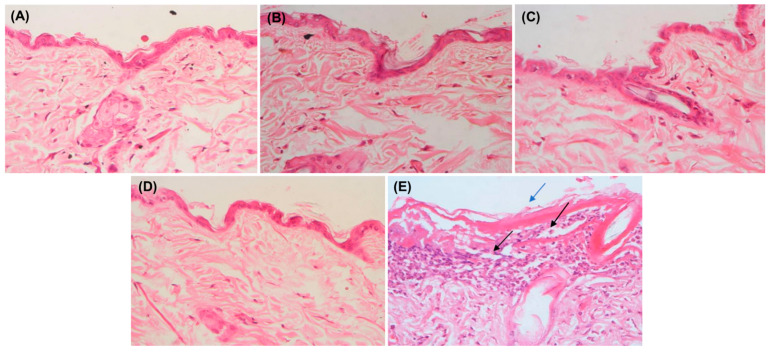
Histopathology of skin sample after skin irritation studies. (**A**) Group I—untreated group. (**B**) Group II—FDG (200 mg from 0.05% gel). (**C**) Group III—CF018 emulgel (200 mg from 0.05% gel). (**D**) Group IV—CF018P emulgel (200 mg from 0.05% gel). (**E**) Group V—received 5% SLS gel [Note (→) Indicates hyperkeratosis (→) Necrosis].

After 72 h, the animals were sacrificed, and the treated skin patches were examined for histopathology, as shown in Figure 12. The epidermis layer of the untreated skin was thin, and no necrosis was observed. It also appeared similarly in CF018 emulgel, CF018P emulgel, and FDG. Increased thickness (hyperkeratosis) of the epidermis layer with necrosis was observed in the case of 5% SLS gel when compared to other formulation treated skin. Because of the binding of the SLS-loaded gel to the keratin in the subcutaneous (SC), ionic and hydrophobic interactions cause the SC to swell. Lipid removal from the SC exposes more keratin binding sites, causing protein denaturation. Surfactants also interact with horny layer proteins to cause reversible skin damage [50]. Lipid removal irreversibly destroys SC structure. CF018 emulgel used non-ionic lipids and surfactants. They do not interact ionically with skin ionic proteins, reducing irritancy. Thus, topical CF018 emulgel is a non-irritant.

#### 3.16.2. FCA-Induced Arthritis Model (Chronic Inflammatory Model)

**Figure 13:**
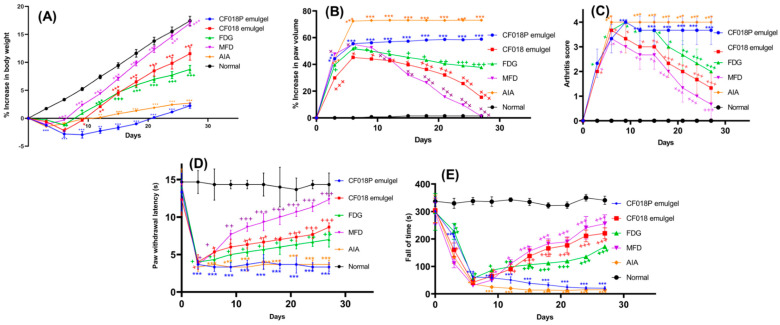
Effect of different formulations on: (**A**) body weight of the rat; (**B**) paw volume of the rat; (**C**) arthritis score; (**D**) paw withdrawal threshold; (**E**) motor incoordination. *** *p* < 0.001. ** *p* < 0.01 vs. normal group; ^+++^ *p* < 0.001. ^++^ *p* < 0.01, ^+^ *p* < 0.5 vs. AIA control.

**Figure 14:**
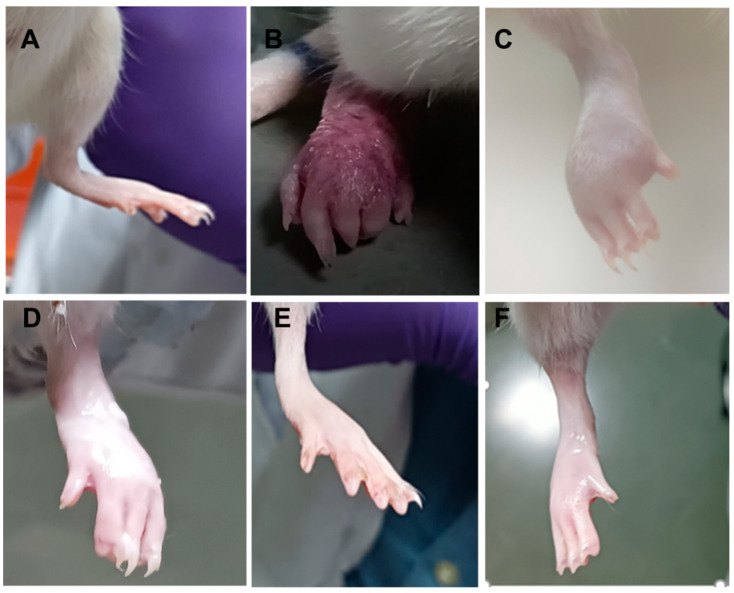
Representative images of FCA injected hind paws after 28 days of treatment with various formulations: (**A**) normal; (**B**) AIA; (**C**) CF018P emulgel; (**D**) FDG; (**E**) CF018 emulgel; (**F**) MFD.

The chronic inflammatory model was developed, and representative results are detailed below, with relevant images depicted in Figure 13 and Figure 14.

##### Assessment of Arthritis from Physical Parameters

During the FCA immunization experiment period, rats lost significant weight (*p* < 0.05–*p* < 0.001), as depicted in Figure 13A. Meanwhile, significant weight gains (*p* < 0.001) were observed in animals treated with MFD, CF018 emulgel, and FDG after the 27th day. The CF018 emulgel resulted in a significant (*p* < 0.5) improvement in body weights when compared to topical FDG administration; however, a similar difference was observed in the MFD-treated group. The results showed that the CF018 emulgel formulation was more effective, with a significant weight gain beginning on the 12th day (*p* < 0.05). On the fifth day, FCA-injected hind paws swelled to maximum and converted to red (first phase). After that, the swelling subsided until the 10th day. Disseminated arthritis caused the paw to swell again (second phase), worse than before (first phase). Topical CF018 emulgel and MFD from the 5th day of arthritis induction suppressed (*p* < 0.01) the second phase (Figure 13B) and reduced hind paw edema/redness in the arthritis groups. As depicted in Figure 13C and Figure 14, the arthritic clinical score decreased significantly from day 15–27 (*p* < 0.05), indicating anti-arthritic activity. Compared to the AIA control group, the blank formulation did not affect body weight change, paw swelling, or arthritic score.

##### Nociceptive Threshold

FCA-induced arthritis, which causes chronic hypersensitivity to nociceptive stimulation, is a good model for the assessment of chronic pain in rats [61]. FCA-treated rodents had lower paw withdrawal thresholds than normal control group animals, and the pain threshold was lowest on day 5, as shown in Figure 13D. Compared to FCA-treated control group animals, CF018 emulgel and MFG reduced withdrawal latency from 1st to 5th day and increased paw withdrawal threshold from the 10th day. As per the results of the hot plate test, which showed an increased reaction time, CF018 emulgel appears to have an anti-nociceptive effect.

##### Motor Incoordination Test

The AIA model causes both pain and motor dysfunction, which ultimately results in hyperalgesia [62]. Motor incoordination is a sign of functional impairment and nervous system inflammation [63]. Motor incoordination was assessed by the mean fall-off time in the rota rod test. FCA sub-plantar injection diminished fall-off time compared to the normal control group. Compared to the FCA control group, rats treated with CF018 emulgel and MFD had a significantly longer fall off time from day 5 to day 28 (Figure 13E). These findings suggest that DTB-induced counter-regulation of spinal-glial activation improved motor coordination in CF018 emulgel. Therefore, DTB-loaded nano-emulgel applied topically may treat arthritis-induced motor-incoordination.

## 4. Outcomes of the Research 

In this research, emulgel was used to improve permeation while minimizing the systemic absorption of DTB, resulting in enhanced efficacy in RA treatment. The QbD method was used to examine the effect of different factors and their interactions in order to achieve the desired product. The higher drug solubility in lipid increased the % EE while decreasing drug leakage from the formulation. The optimal concentration of Smix in the formulation favored low PS with even distribution by stabilizing the oil-in-water interface. Carbopol ETD 2020 exhibited excellent spreadability and consistency during storage. The prepared CF018 emulgel spread as a thin layer on the skin’s surface, hydrating it by reducing trans-epidermal water loss. This hydration promotes skin lipid swelling and helps to improve permeation. Ex vivo studies revealed that the CF018 emulgel had better permeation than FDG. Further, in vivo studies revealed that the CF018 emulgel exhibited better efficacy in FCA induced rat model. The study demonstrated the ease of developing emulgel formulations from a wide range of materials, with improved safety and efficacy.

## 5. Conclusions

In this study, the improvement in skin permeation and deposition of a DTB-loaded emulgel was determined using a QbD-based approach. Using a CCD, the effect of lipid, Smix concentration, and homogenization time on PS and % EE was investigated. The results revealed that topical DTB-loaded emulgel increased skin permeation and deposition, as expected, due to the occlusive effect of forming a very thin layer on the skin’s surface. The emulsion’s in vitro drug release demonstrated sustained release. The cell culture results illustrated the safety of the excipients, and the decrease in TNF-α levels expressed the efficacy of the CF018 emulgel formulation. As a result, we conclude that topical delivery of DTD-loaded nano emulgel is a viable approach for the treatment of RA. However, more studies are required to improve the DTB loading to simulate the clinical dose for humans in the treatment of RA.

## Figures and Tables

**Table 1:** REM for initial risk assessment and FMEA score based on qualitative analysis of different material attributes and process parameters.

CMAs and CPPs	CQAs			FMEA			
	PS	EE	Drug Release	Severity	Detectability	Occurrence	RPN Score
Amount of Lipid	HIGH	HIGH	HIGH	8	6	6	288
Smix concentration	HIGH	HIGH	HIGH	8	5	6	240
Co-solvent	MEDIUM	MEDIUM	MEDIUM	5	3	4	60
Permeation enhancer	MEDIUM	MEDIUM	MEDIUM	5	3	4	60
Homogenization Time	HIGH	HIGH	LOW	6	5	5	150
Homogenization speed	HIGH	HIGH	LOW	7	5	5	175
Process temperature	LOW	MEDIUM	LOW	4	3	2	24

**Table 2:** Experimental trails executed using CCD with respective results.

Formulation Code	Factor1 A: Lipid Base (%)	Factor 2 B: Smix (%)	Factor 3 C: Homogenization Speed (RPM)	Factor 4 D: Homogenization Time (min)	Response 1 Size (nm)	Response 2 EE (%)	PDI
CF1	5	3	10,000	15	186.03 ± 1.70	87.87 ± 0.16	0.157 ± 0.009
CF2	6	4	15,000	5	310.70 ± 3.63	84.31 ± 0.25	0.358 ± 0.033
CF3	5	4	10,000	10	312.27 ± 2.84	86.30 ± 0.13	0.304 ± 0.010
CF4	4	4	5000	15	222.47 ± 5.48	86.95 ± 0.10	0.294 ± 0.021
CF5	6	2	5000	15	181.30 ± 10.54	84.53 ± 0.13	0.292 ± 0.050
CF6	6	2	15,000	15	193.53 ± 8.0	84.92 ± 0.63	0.343 ± 0.023
CF7	4	4	15,000	15	163.77 ± 3.93	91.21 ± 0.10	0.231 ± 0.012
CF8	4	2	5000	5	271.23 ± 47.89	88.64 ± 0.44	0.416 ± 0.114
CF9	6	4	5000	5	1782.53 ± 770.61	95.23 ± 0.24	0.952 ± 0.083
CF10	6	3	10,000	10	445.13 ± 147.90	90.09 ± 0.05	0.549 ± 0.135
CF11	5	3	5000	10	374.37 ± 2.87	91.02 ± 0.06	0.348 ± 0.032
CF12	5	3	10,000	10	308.77 ± 2.15	89.81 ± 0.18	0.284 ± 0.033
CF13	5	3	15,000	10	275.77 ± 4.44	89.71 ± 0.31	0.282 ± 0.027
CF14	5	3	10,000	5	322.90 ± 1.35	91.85 ± 0.42	0.273 ± 0.029
CF15	4	2	15,000	5	300.37 ± 1.50	86.53 ± 0.21	0.244 ± 0.011
CF16	5	2	10,000	10	314.67 ± 2.24	84.38 ± 0.61	0.317 ± 0.023
CF17	4	3	10,000	10	297.40 ± 3.48	93.30 ± 0.40	0.313 ± 0.045

**Table 3:** Release kinetics data of DTB-loaded emulsion.

Batch Size (Ml)		Zero Order	First Order	Higuchi	Korsmeyer Peppas	Hixson Crowell
20	R^2^	0.6900	0.9790	0.9430	0.9454	0.9912
20	AIC	75.6555	51.4332	60.4207	62.0220	43.5941
50	R^2^	0.7266	0.9595	0.9064	0.9163	0.9710
50	AIC	73.6516	56.4597	64.0006	64.9945	53.4487
100	R^2^	0.6329	0.9733	0.9239	0.9243	0.9809
100	AIC	76.2149	52.6162	62.0542	64.0076	49.5992

## Data Availability

Not applicable.

## Supplementary Materials

The following supporting information can be downloaded at: https://www.mdpi.com/article/10.3390/pharmaceutics15030736/s1, Table S1: Experimental trails performed using two-level factorial design. Table S2: The QTPP of API loaded emulgel. Table S3: CQA’s of API loaded Emulgel. Table S4: The percent contribution of the factors and their responses. Table S5: Responses of experimental trails performed using two-level factorial design. Table S6: Comparative in vivo skin irritation studies between CF018P emulgel, CF018 emulgel, FDG & 5% SLS Gel. Figure S1: Solubility of DTB in different Oils and Surfactants. Figure S2: Pareto chart representation for two-level factorial design depicting the interaction and efficiency of the factors against the responses (A) Size; (B) Entrapment; (C) Drug release. Figure S3: ATR-IR Peaks of (A) Dasatinib (B) CF018P emulgel (C) CF018 emulgel.
