# Correction: Donthi et al. Dasatinib-Loaded Topical Nano-Emulgel for Rheumatoid Arthritis: Formulation Design and Optimization by QbD, In Vitro, Ex Vivo, and In Vivo Evaluation. *Pharmaceutics* 2023, *15*, 736

**DOI:** 10.3390/pharmaceutics17040525

**Published:** 2025-04-16

**Authors:** Mahipal Reddy Donthi, Ranendra Narayan Saha, Gautam Singhvi, Sunil Kumar Dubey

**Affiliations:** Department of Pharmacy, Birla Institute of Technology and Science, Pilani (BITS-PILANI), Pilani Campus, Pilani 333031, Rajasthan, India

## Error in Figure

In the original publication [1], there was a mistake in Figure 11 as published. In this study, the 72-h time point is considered after treatment. To maintain transparency, we included images from several time points which led to inadvertent duplication during the image combining process. Specifically, in each cohort, the 0-h time point includes two images (pre- and post-formulation), and for Group I (negative control, zero hour), no formulation was applied, hence the images look similar. Additionally, we noticed a similarity between the 24-h image of Group II and that of Group III, likely due to our error in arranging the images. However, as detailed in Section 3.16.1 of the manuscript, our conclusions are based on histopathological results at the 72-h mark, with earlier time points serving only for preliminary visual inspection. The corrected Figure 11 appears below.

The authors state that the scientific conclusions are unaffected. This correction was approved by the Academic Editor. The original publication has also been updated.

## Figures and Tables

**Figure 11:**
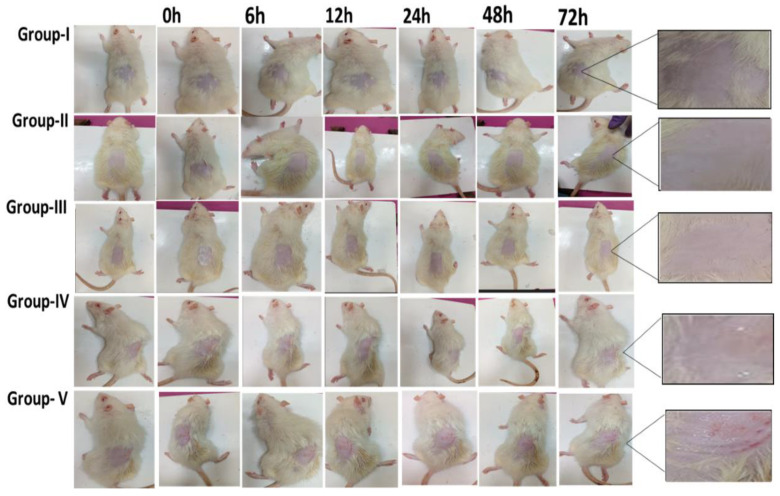
In vivo skin irritation study of Group I—untreated group; Group II—FDG (200 mg from 0.05% gel); Group III—CF018 emulgel (200 mg from 0.05% gel); Group IV—CF018P emulgel (200 mg from 0.05% gel); Group V—received 5% SLS gel.
