# Supplementary material for: Dasatinib-Loaded Topical Nano-Emulgel for Rheumatoid Arthritis: Formulation Design and Optimization by QbD, In Vitro, Ex Vivo, and In Vivo Evaluation
Source: Pharmaceutics. 2023 Feb 22;15(3):736. doi: 10.3390/pharmaceutics15030736 (PMC10052882; doi:10.3390/pharmaceutics15030736)
Supplement: Supplementary file 1 [file pharmaceutics-15-00736-s001.zip › pharmaceutics-2153020-supplementary.pdf]

Supplementary Table S1: Experimental trails performed using two-level factorial design.

Ingredients (g)	DF1	DF2	DF3	DF4	DF5	DF6	DF7	DF8
**API**	0.01	0.01	0.01	0.01	0.01	0.01	0.01	0.01
**Peceol +Geleol (7:3)**	1	1	4	4	1	1	4	4
**Labrasol + capryol (2:1)**	2	0.6	0.6	2	0.6	2	0.6	2
**Transcutol P**	2	2	2	0.5	0.5	0.5	0.5	2
**Isopropyl myristate**	0.5	2	0.5	0.5	0.5	2	2	2
**Methyl paraben**	0.1	0.1	0.1	0.1	0.1	0.1	0.1	0.1
**Propyl paraben**	0.01	0.01	0.01	0.01	0.01	0.01	0.01	0.01
**Sodium metabisulfite**	0.04	0.04	0.04	0.04	0.04	0.04	0.04	0.04
**Aqueous phase**	14.5	14.4	12.9	13	17.4	14.5	12.9	10
**Total weight (g)**	20	20	20	20	20	20	20	20

Supplementary Table S2: The QTPP of API loaded emulgel.

Quality Target Product profile	Target	CQAs	Justification
**Dosage form**	Emulgel	NA	To enhance the permeation of API
**Route of administration**	Topical	NA	Enhance the DTB concentration at targeted site without systemic side impacts
**Dosage strength**	0.05%	NA	To select the minimum effective dose
**Stability**	At least 3 months stability at room temperature	Yes	Effect the product quality
**Particle size (nm)**	< 200	Yes	Enhance the drug permeation through skin barriers
**Polydispersity index (PDI)**	< 0.3	Yes	perfectly uniform size distribution leads to uniform release, permeation, and drug loading in nanoparticles
**% Entrapment efficiency (% EE)**	>90	Yes	Greater entrapment leading to higher drug loading with less lipid concentration
***In vitro* drug Release profile**	Sustained	Yes	Increased activity time without systemic exposure

**pH**	5.5	Yes	Affect the physiochemical stability and mimic with physiological pH leads to reduce the skin irritation
**Solubility**	Maximum	Yes	Impact the drug permeation

Supplementary Table S3: CQA's of API loaded Emulgel.

Critical Quality Attributes	Related to CMAs	Related to CPPs	Failure mode	Justification
**Particle size (PS)**	Concentration of Smix and lipid	Change in homogenization speed and time	Entrapment, loading efficiency, drug release and skin permeation of the drug	• Increase in concentration of lipid increases size resulting in decrease in release and permeation<br>• Increase in speed and time of homogenization and concentration of Smix decreases size, entrapment and loading efficiency
**Entrapment efficiency (EE)**	Concentration of Smix and lipid	Change in homogenization speed and time	loading efficiency, Size, drug release and skin permeation	• Increase in concentration of lipid andSmix increases entrapment resulting in decrease in drug release and permeation (due to lipid portion effect)<br>• Increase in speed and time of homogenization decreases entrapment, loading efficiency
**Drug release**	Concentration of Smix and lipid	Change in homogenization speed and time	Poor therapeutic effect	• Increase in concentration of lipid and Smix increases size and lipid matrix resulting in decrease in drug release and permeation<br>• Increase in speed and time of homogenization increases release due to the decreased size (indirect effect)

Supplementary Table S4: The percent contribution of the factors and their responses

Code	Name of the factor	Contribution on size (%)	Contribution on entrapment (%)	Contribution on drug release (%)
**A**	Lipid	50.31	31	84.77
**B**	Surfactant	11.81	41.77	3.29
**C**	Isopropyl myristate	0.07	0.042	0.057
**D**	Transcutol -P	6.76	3.82	1.23
**AB**	Lipid + Surfactant	26.26	15.91	3.22
**AC**	Lipid + Isopropyl myristate	0.093	7.44	7.16
**AD**	Lipid + Transcutol -P	NC	0.02	0.26
**BC**	Surfactant + Isopropyl myristate	NC	NC	NC
**BD**	Surfactant + Transcutol -P	NC	NC	NC
**CD**	Isopropyl myristate + Transcutol -P	NC	NC	NC
**ABC**	Lipid + Surfactant + Isopropyl myristate	NC	NC	NC
**ABD**	Lipid + Surfactant + Transcutol -P	NC	NC	NC
**ACD**	Lipid + Isopropyl myristate + Transcutol -P	NC	NC	NC
**BCD**	Surfactant + Isopropyl myristate + Transcutol -P	NC	NC	NC
**ABCD**	Lipid + Surfactant+ Isopropyl myristate + Transcutol -P	NC	NC	NC

*** NC - No contribution**

Supplementary Table S5: Responses of experimental trails performed using two-level factorial design

Formulation code	Size (nm)	% Entrapment	% Drug release (24h)	PDI
**DF1**	546.97 ± 48.9	96.25 ± 0.5	69.80 ± 3.30	0.401 ± 0.02
**DF2**	311.70 ± 19	88.44 ± 0.21	86.30 ± 1.80	0.280 ± 0.01
**DF3**	2055.00 ± 19	92.70 ± 0.23	55.10 ± 3.60	0.380 ± 0.07
**DF4**	559.00 ± 80	93.32 ± 0.11	46.97 ± 2.50	0.655 ± 0.03
DF5	223.00 ± 15.13	89.97 ± 0.18	90.58 ± 3.30	0.141 ± 0.07
**DF6**	620.00 ± 21.35	92.28 ± 0.23	84.00 ± 3.00	0.883 ± 0.16
**DF7**	3054.00 ± 40	91.50 ± 0.32	49.10 ± 6.50	0.412 ± 0.05
**DF8**	1345.00 ± 380	91.28 ± 0.53	57.20 ± 5.80	0.724 ± 0.06

Supplementary Table S6: Comparative *in vivo* skin irritation studies between CF018P emulgel, CF018 emulgel, FDG & 5% SLS Gel.

**Erythema and Oedema**					
**Time (h)**	**CF018P emulgel**	**CF018 emulgel**	**FDG**	**5% SLS GEL**	**Normal group**
0	0	0	0	0	0
1	0	0	0	0	0
6	0	0	0	0	0
12	0	0	0	1	0
24	0	0	0	2	0
48	0	0	0	3	0
72	0	0	1	3	0
**Atonia- A decrease in normal elasticity of the skin**					
0	1	1	1	1	1
1	1	1	1	1	1
6	1	1	1	2	1
12	1	1	1	2	1
24	1	1	1	3	1
48	1	1	1	3	1
72	1	1	2	3	1
**Fissuring- Cracks on skin**					
0	1	1	1	1	1
1	1	1	1	1	1
6	1	1	1	1	1
12	1	1	1	1	1
24	1	1	1	2	1
48	1	1	1	2	1
72	1	1	1	2	1

Note: Grading for skin reaction for Erythema and Oedema (0- None, 1- Slight, 2- Moderate, 3- Severe), Atonia - A decrease in normal elasticity of the skin (1- Slight (slight impairment of elasticity, 2- Moderate (slow return to normal), 3- Marked (No elasticity)), Fissuring - Cracks on skin (1- Slight (Definite cracks in epidermis), 2- Moderate (Cracks on dermis), 3- Marked (Cracks and bleeding).

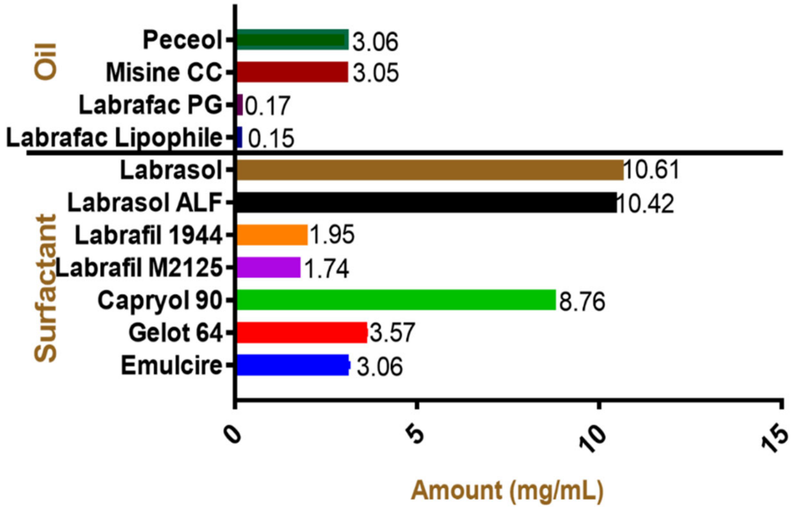

Supplementary Figure F1: Solubility of DTB in different Oils and Surfactants

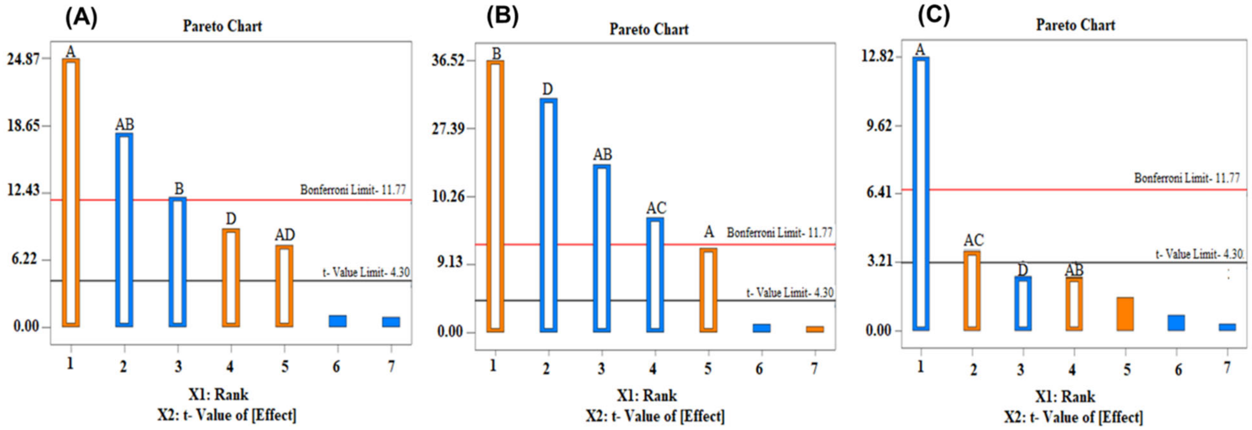

Supplementary Figure F2: Pareto chart representation for two-level factorial design depicting the interaction and efficiency of the factors against the responses (A) Size; (B) Entrapment; (C) Drug release

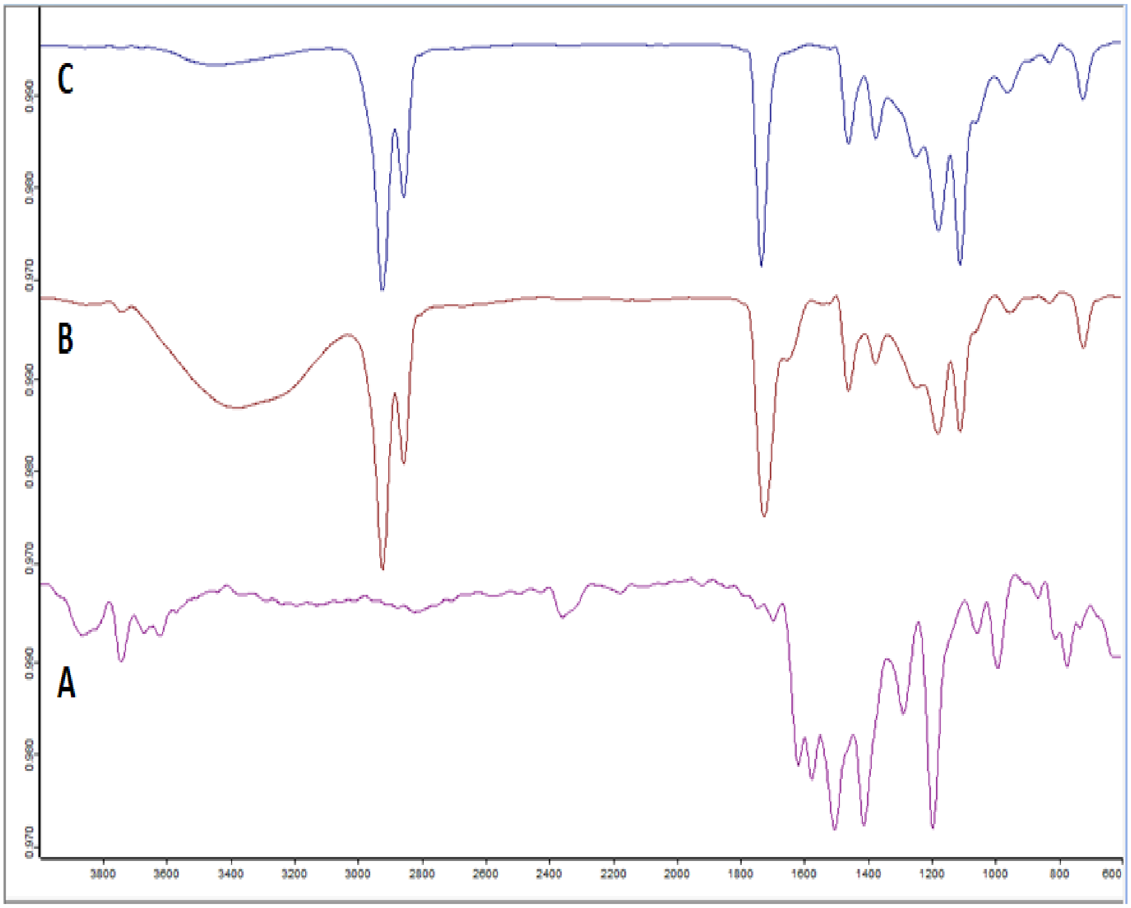

Supplementary Figure F3: ATR-IR Peaks of A) Dasatinib B) CF018P emulgel C) CF018 emulgel
